# Graphene Oxide Membranes for Tunable Ion Sieving in Acidic Radioactive Waste

**DOI:** 10.1002/advs.202002717

**Published:** 2021-02-18

**Authors:** Tong Wu, Zhe Wang, Yuexiang Lu, Shuang Liu, Hongpeng Li, Gang Ye, Jing Chen

**Affiliations:** ^1^ Institute of Nuclear and New Energy Technology (INET) Collaborative Innovation Center of Advanced Nuclear Energy Technology Tsinghua University Beijing 100084 P. R. China; ^2^ The MOE Key Laboratory of Resource and Environmental System Optimization School of Environment and Chemical Engineering North China Electric Power University Beijing 102206 P. R. China

**Keywords:** graphene oxide membrane, ions sieving, separation, uranium

## Abstract

Graphene oxide (GO) membranes with unique nanolayer structure have demonstrated excellent separation capability based on their size‐selective effect, but there are few reports on achieving ion–ion separation, because it is difficult to inhibit the swelling effect of GO nano sheets as well as to precisely control the interlayer spacing *d* to a specific value between the sizes of different metal ions. Here, selective separation of uranium from acidic radioactive waste containing multication is achieved through a precise dual‐adjustment strategy on *d*. It is found that GO swelling is greatly restricted in highly acidic solution due to protonation effect. Then the interlayer spacing is further precisely reduced to below the diameter of uranyl ion by increasing the oxidation degree of GO. Sieving uranyl ions from other nuclide ions is successfully realized in pH =3–3 mol L^−1^ nitric acid solutions.

## Graphical abstract

Precisely controlling the interlayer spacing of graphene oxide (GO) membrane in a wide range (11.4–15.5 Å) is realized by using a dual‐adjustment strategy of combining acidity with oxidation degree. Such GO membranes can successfully sieve uranyl ions from other nuclide ions in pH =3–3 mol L^−1^ HNO_3_ aqueous, showing their potential application in the treatment of highly acidic radioactive waste.

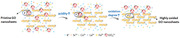

## 1 Introduction

Graphene oxide (GO) membranes have attracted widespread attention in the separation of molecules and ions in the solution due to its unique size sieving effect, which is based on the interlayer spacing (*d*) between GO nanosheets. By tuning the interlayer spacing to a specific range, the GO membranes have been successfully used in the separation between biomolecules/solvent,^[^ ^1^ ^]^ dye/metal ions,^[^ ^2^, ^3^, ^4^, ^5^ ^]^ organic solvent/solvent,^[^ ^6^, ^7^ ^]^ ions/solvent^[^ ^8^, ^9^ ^]^ and et al. In particular, the prospect of using the GO membrane to realize the separation of ions with varying size is fascinating,^[^ ^10^, ^11^, ^12^, ^13^, ^14^ ^]^ as it has many promising applications such as mining/recycling of valuable metal, removal/extraction of specific toxic and harmful metal ions. However, it is fairly difficult to hit this target because of two reasons. First, the interlayer spacing of GO nano sheets would tend to swell in aqueous solution (*d* ≈ 13.5 Å) which is far larger than the diameter of common hydrated ions,^[^ ^15^ ^]^ resulting in the failure to intercept these ions.^[^ ^3^, ^16^, ^17^, ^18^ ^]^ Second, as the size difference among various metal ions is usually less than several angstroms, it requires precise controlling of the interlayer spacing to a specific value within atomic range, making it extremely challenging to achieve desirable ion–ion selectivity. Up to now, only a few ingenious strategies have been proposed to limit the swelling effect and realized accurate tuning of *d* to a value between the sizes of target metal ions, such as electrochemical treatment,^[^ ^4^ ^]^ molecules/ions cross‐linking,^[^ ^10^ ^]^ photon‐electron‐ion transport phenomenon,^[^ ^11^ ^]^ and physical confinement.^[^ ^12^ ^]^ It is still highly required to develop simple and feasible modulate methods to regulate the interlayer spacing and achieve ion–ion separation, especially for a specific practical application.

Uranium‐based fission nuclear energy, as a kind of important clean energy, has potential to partially replace fossil fuels energy.^[^ ^19^, ^20^, ^21^ ^]^ While, during the production, utilizing, and reprocessing of nuclear fuel, plenty of radioactive wastewater will be generated, which is a kind of specific acidic mixed metal ions system containing UO_2_^2+^.^[^ ^22^ ^]^ In consideration of the shortage of uranium resource on the earth as well as the chemical toxicity of uranium,^[^ ^23^ ^]^ it is necessary to extract/remove uranium from the radioactive waste liquid for recycling and utilizing uranium as well as protecting the human health and the environment.^[^ ^24^, ^25^, ^26^, ^27^ ^]^ However, due to the extreme properties of the wastewater such as high salty (≈400 g L^−1^), high acidity (≈3 mol L^−1^), and high radioactivity (>3.7 × 10^10^ Bq L^−1^), it is still a huge challenge to develop suitable methods and materials for separating uranium from other metal ions.^[^ ^28^ ^]^ Up to now, solvent extraction^[^ ^29^ ^]^ is the only method used for large‐scale industrial application. However, it still has some disadvantages, such as the radiolysis of organic extraction agents would lower the extraction efficiency and large amount of secondary waste would increase the waste volume and as well as disposal costs. Solid phase extraction^[^ ^30^ ^]^ as an alternative method has been developed rapidly in recent years. Various kinds of extraction materials have been synthesized for this application, but few of them could work under highly acidic condition. Although some adsorption materials with functional groups can selectively combine with uranyl ions under low‐acid conditions (pH 2–7),^[^^]^ the coordination ability of these functional groups would be much weakened when pH < 2 because of the protonation.^[^ ^31^ ^]^ Moreover, these materials themselves usually have poor stability under high‐acid condition and are difficult to be applied. Graphene oxide as a kind of carbon material has shown excellent stability under acidic and radioactive conditions,^[^ ^32^, ^33^ ^]^ but binding the metal ions with the abundant functional groups on GO surface will also be greatly inhibited in such condition. Utilizing the size sieving effect instead of chemical adsorption may provide another way for the separation application.

Herein, we report selective separation of uranium from other metal ions in highly acidic multication solution by using GO membrane with precisely adjusted interlayer spacing. We found that high‐acid condition could restrain the swelling of GO membrane, resulting in a narrowed interlayer spacing, which is close to the diameter of uranyl ion. In addition, by increasing the oxidation degree of GO nano sheets, the interlayer spacing could be further reduced. Through this dual‐adjustment strategy, the interlayer spacing can be accurately controlled in a wide range (15.5–11.4 Å), and sieving uranyl ions from other nuclide ions was successfully realized in pH = 3–3 mol L^−1^ nitric acid solutions. Our work demonstrate that the GO membrane has potential to be applied in the field of metal ions separation in radioactive liquid waste. Moreover, the dual control strategy of combining acidity with oxidation degree offers a simple and convenient route to modulate the interlayer spacing, which might be useful in other application areas.

## 2 Results and Discussion

### 2.1 Sieving Performance of GO Membranes in Highly Acidic Solution

**Figure 1:**
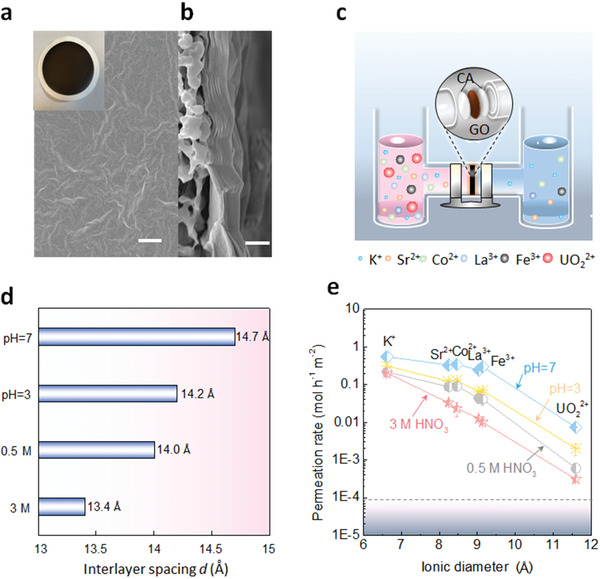
The morphology of GO membrane and ionic permeation tests. a) Scanning electron microscope image of GO membrane surface (insert: digital photo of GO membrane). Scale bar, 20 µm. b) Morphology of GO membrane in cross section. Scale bar, 1 µm. c) Schematic of a H‐shaped glass filter with two compartments (feed and permeate) used in permeation tests. The feed side includes mixed 80 mL KNO_3_, Sr(NO_3_)_2_, Co(NO_3_)_2_, La(NO_3_)_3_, Fe(NO_3_)_3_, and UO_2_(NO_3_)_2_ solutions with various acidity, and the permeate side includes 80 mL corresponding nitric acid solutions. Cellulose acetate (CA) membrane is used as the support and the effective diameter of GO membrane is 1.6 cm. Magnetic stirring is used to avoid concentration generation. d) The interlayer spacing *d* of GO membranes (≈800 nm) which soaked in aqueous solution with various acidity (pH = 7, pH = 3, 0.5, and 3 mol L^−1^) for 5 h. e) Ion permeation tests of the GO membrane (≈800 nm) in nitric acid with different concentration after 5 h. Gray area stands for the below‐detection limit of the ICP‐OES.

GO suspension was prepared by modified two‐step Hummers method and then exfoliated by sonication. After that, GO membranes were fabricated from GO suspension through vacuum filtration. The thickness of membrane can be controlled through ensuring the suspension volume. Scanning electron microscope (SEM) images manifested that the surface of GO membrane has a large number of wrinkles (**Figure** **1**a). The cross section of GO membrane showed a typical layer structure (Figure 1b), and it is also found there are some wrinkles in GO laminates (Figure S1, Supporting Information), which could improve the permeance of metal ions.

The obtained GO membrane was firstly soaked in aqueous solutions with different acidity (pH = 7, pH = 3, 0.5, and 3 mol L^−1^) for 5 h and it was found the GO membrane in water could not maintain its structure because of the swelling effect (Figure S2, Supporting Information), showing that the membrane had inferior chemical stability and separation quantity when combining with considerable water molecules. While, interestingly, after soaked in acidic solution for 5 h, the GO membranes were still stable. The interlayer spacing *d* dramatically reduced from 14.7 to 13.4 Å with the acidity increasing from pH = 7 to 3 mol L^−1^ (Figure 1d). This decline can be ascribed to the weakened electrostatic force between the GO sheets which was caused by the protonation of ionized carboxyl groups (COO^−^) in GO. Zeta potential of GO membrane was changed from −39.6 to −29.5 mV when the acidity increased from pH = 7 to 3 mol L^−1^ HNO_3_ (Figure S3, Supporting Information).

By taking advantage of the tunability of interlayer spacing in acidic condition, the permeation behaviors of GO membranes in various acidic solutions were investigated. For permeation experiments, the GO membranes were fixed in the middle of an H‐shaped glass filter, which comprised of feed and permeate compartments (Figure 1c). The feed compartment was filled with the mixture solution of K^+^, Sr^2+^, Co^2+^, La^3+^, Fe^3+^, and UO_2_^2+^ (the concentration of each cation in the feed side is 0.05 mol L^−1^ while the concentration of UO_2_^2+^ is 0.001 mol L^−1^) at the presence of the nitric acid concentration with pH = 7, pH = 3, 0.5, and 3 mol L^−1^, respectively, while the permeate compartment was only filled with the same volume of corresponding nitric acid. Figure 1d showed that the permeability of metal ions was related to their sizes, and the reduced *d* caused by increasing acidity promoted the ions separation performance. On account metal cations are difficult to combine NO_3_^−^ ions in acidic solution,^[^ ^34^ ^]^ we can assume that they occupy the volume given by the hydrated diameter. The permeate rates of K^+^ (6.62 Å), Sr^2+^(8.24 Å), and UO_2_^2+^ (11.6 Å)^[^ ^35^ ^]^ reduced to about 2.5, 10.1, and 23.5 times separately when acidity increasing from pH = 7 to 3 mol L^−1^ HNO_3_. In the case of 3 mol L^−1^ HNO_3_, K^+^ had the fastest permeation rate of 0.15 mol m^−2^ h^−1^, while UO_2_^2+^ showed the slowest permeated rate of 2 × 10^−4^ mol m^−2^ h^−1^. These results indicated that GO membrane had the potential to selectively sieve various ions in highly acidic solution. However, the initial prepared GO membrane was failed to achieve the complete interception of uranyl ions by increasing the acidity. Indeed, the effective nano channels *μ* can be calculated as *d*  −  *ε*  , where *ε*≈ 3.4 Å is the thickness of monolayer GO.^[^ ^12^, ^36^, ^37^ ^]^ Although the GO membrane had *μ* of 13.4 − 3.4 = 10.0 Å in 3 mol L^−1^ HNO_3_, the transport of some uranyl ions (11.6 Å) was permitted due to the dehydration effect.^[^ ^12^, ^38^ ^]^ In this case, the hydration shells of ions will partially remove the water molecules for reducing their size to fit into GO nano channels. Therefore, a precise adjustment of the interlayer spacing to smaller value was required to obtain a preferable UO_2_^2+^ sieving performance.

### 2.2 Synthesis and Characterization of Four Kinds of GO with Different Oxidation Degrees

To further precisely control the interlayer spacing *d* to intercept uranium ions, four kinds of GO nano sheets with different oxidation degrees were synthesized. These GO nano sheets were prepared by changing the duration of middle (35 °C) and high (90 °C) temperature reactions, and they were nominated as GO‐M1 (35 °C for 1 h), GO‐M2 (35 °C for 2 h), GO‐H1 (35 °C for 2 h and 90 °C for 10 min), and GO‐H2 (35 °C for 2 h and 90 °C for 30 min). Notice that GO‐M2 was the above mentioned initial GO. It was reported that prolonging the middle and high temperature reactions was contributed to increasing the oxidation degrees, which was related to the number of oxygen‐containing functional groups and the size of GO nano sheets.

**Figure 2:**
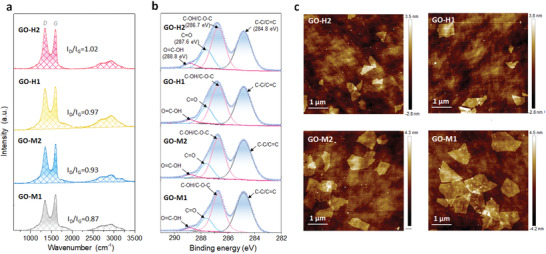
Characterization of GO membranes with different oxidation degrees. a) Raman spectra of four kinds of GO membranes with different oxidation degrees. b) C1s XPS spectra of four kinds of GO samples. c) Atomic force microscope (AFM) images of the four kinds of GO nano sheets on the silicon substrate. Scale bar, 1 µm.

The intensities ratio of Raman peaks D and G (*I*_D_ / *I*_G_) were used to estimate the integrity of GO nano sheets (**Figure** **2**a). The larger the *I*_D_ / *I*_G_, means the more defects were generated. *I*_D_ / *I*_G_ increases with the middle and high temperature reaction time extending, from 0.87 (GO‐M1), to 0.93 (GO‐M2), 0.97 (GO‐H1) and 1.02 (GO‐H2). The D’ peak (1620 cm^−1^) in four samples was negligible suggesting that the sp3‐like defects were the main type of the defects instead of vacancy‐type defects.^[^ ^39^ ^]^ FT‐IR spectra (Figure S4, Supporting Information) was used to detect the functional groups. All four samples had peaks at 1650, 3400, 1250, and 1600 cm^−1^, which can be assigned to the carboxyl groups, hydroxyl groups, ether groups, and unoxidized sp2 C—C bonds. GO‐H2 showed the strongest absorption intensities at 1650 and 3400 cm^−1^, implying it had the largest amount of carboxyl and hydroxyl groups and the highest oxidation degree. In the meanwhile, X‐ray photoelectron spectrometer (XPS) (Figure 2b) was also used to detect the functional groups species. C1s spectrum of all four samples can be divided into typical peaks corresponding to carbon–carbon and carbon–oxygen bonds: C—C/C=C (284.8 eV), C—O (286.7 eV), C=O (287.6 eV), and O=C—OH (288.8 eV).^[^ ^6^, ^40^ ^]^ The ratio of the O/C also can demonstrate the oxidation degree (Figure S5, Supporting Information). From GO‐M1 to GO‐H2, the ratios of O/C increased, implying the increase of the oxygen‐containing groups. Furthermore, the higher oxidation degree (e.g., GO‐H2) can lead to smaller GO nanosheets size (Figure 2c and Figure S6, Supporting Information) and make them easier to be exfoliated into single layers under ultrasonic conditions.^[^ ^41^, ^42^ ^]^ As the oxidation degree increasing, the average size of GO sheets gradually reduced from 0.82 µm (GO‐M1) to 0.49 µm (GO‐M2), 0.32 µm (GO‐H1), and 0.19 µm (GO‐H2).

### 2.3 Sieving Performance of Four GO Membranes in 3 mol L^−1^ Nitric Acid Solution

**Figure 3:**
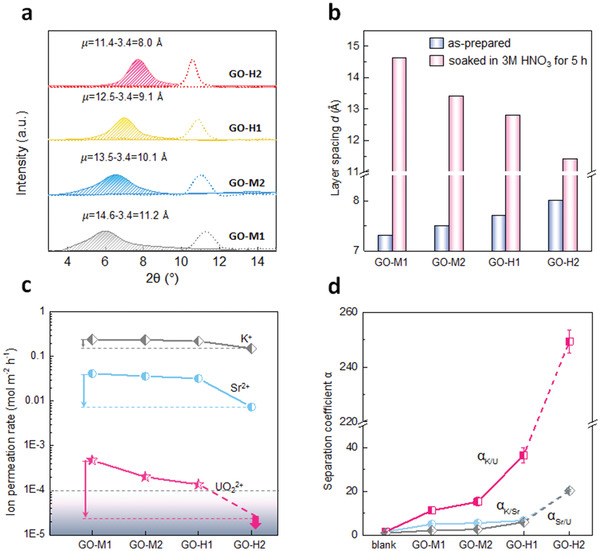
XRD pattern of GO membranes with different oxidation degrees and corresponding ion permeation tests. a) XRD patterns of four GO membranes before (dashed line) and after (solid line) soaked in 3 mol L^−1^ nitric acid for 5 h. b) Interlayer spacing of four GO membranes before and after soaking in 3 mol L^‐1^ HNO_3_. c) Ion permeation rates of K^+^, Sr^2+^, UO_2_^2+^ on GO membranes with different oxidation degrees. Gray area stands for the below‐detection limit of our measurements with the arrow indicating the detection limit of metal ions. d) Separation coefficient *α* of K^+^ and UO_2_^2+^, Sr^2+^ and UO_2_^2+^, Sr^2+^and UO_2_^2+^ ions for four GO membranes.

Four GO membranes were then fabricated by using these four kinds of GO nano sheets, and named as the same as the origin GO sheets. The thicknesses of all membranes were controlled to be about 800 nm (Figure S7, Supporting Information). The interlayer spacing *d* of GO membranes was measured by XRD pattern (**Figure** **3**a). In the case of dry GO membranes (dashed line in Figure 3a), the *d* (blue column in Figure 3b) expanded from 7.3 to 8.0 Å with the increasing oxidation degree of GO. Such expansion can be ascribed to that GO sheets with higher oxidation degree contains more oxygen‐containing groups, making them more liable to interact with each other through hydrogen bonding and thus induce wrinkles on the GO sheets. The abundant oxygen‐containing groups could also absorb one or two layers of H_2_O molecules during filtration process that will enlarge the *d*.^[^ ^12^ ^]^ Moreover, compared to the large‐sized GO sheets, the small‐sized GO sheets provide more edges with functional groups, resulting in stronger electrostatic repulsion to hinder stacking. As a result, membrane with high oxidation has more wrinkles, looser structure, and larger *d* (Figure 3a and Figure S7, Supporting Information).

When the GO membranes were soaked in 3 mol L^−1^ nitric acid, the interlayer spacing (pink column in Figure 3b) of all membranes increased obviously due to the intercalation of H_2_O molecules, which is well known as swelling effect and the degree of swelling depends on the property of the solvent.^[^ ^6^ ^]^ Interestingly, compared to that in dry condition, the interlayer spacing of four GO membranes showed a different change trends. The *d* narrowed from 14.6 (GO‐M1) to 11.4 Å (GO‐H2) with the increase in oxidation degree of GO. It is because there are more functional groups in the edges or in‐plane of GO sheets under high oxidation degree, correspondingly more carboxyl groups (COO^−^) would tend to interact with protons under acidic conditions. The enhanced protonation will gradually weaken the electrostatic force between the GO sheets due to the decline in charge density, leading to the decrease in interlayer spacing.^[^ ^43^, ^44^, ^45^ ^]^

Then the permeation behavior of different ions on these GO membranes was investigated. Figure 3c manifested that with the decrease of interlayer spacing, the permeation rates of K^+^, Sr^2+^, and UO_2_^2+^ ions reduced in different degree. For K^+^ ions (6.62 Å), the permeation rate shows a very slight decline from 0.24 to 0.15 mol m^−2^ h^−1^ with *d* decreasing. Sr^2+^ ions have a larger size of 8.24 Å, thus shows a higher influence by the reduced *d* and its permeation rate decrease about 5 times from 4.2 × 10^−2^ to 7.4 × 10^−3^ mol m^−2^ h^−1^. In the case of UO_2_^2+^ ions (11.6 Å), the permeation rate decrease for about 20 times from 4.7 × 10^−4^ to 1.5 × 10^−5^ mol m^−2^ h^−1^ with the *d* decreased. Especially on the GO‐H2 membrane, a cut‐off blocking of UO_2_^2+^ ions was achieved as the permeated amount was below the detection limit. It can be ascribed to the effective nano channels *μ* of GO‐H2 (11.4−3.4 = 8.0 Å), which is exactly lower than the hydrated diameter of UO_2_^2+^ ions even dehydration effect happened. It is reported that cations also can reduce the interlayer spacing because ions with positively charged will suppress the electric double layer of GO.^[^ ^46^, ^47^ ^]^ But in this research system, the acidity rather than cation's impact played a vital role (Figure S8, Supporting Information). It can be attributed to the relative higher proton concentration and lower ions concentration than that of in other literatures.^[^ ^10^ ^]^

To estimate the separation performance between UO_2_^2+^ and other ions on different GO membranes, and eliminate the influence of initial metal ions concentration, separation coefficient *α*_A/B_ is defined as follows

(1) $$\alpha_{A/B}=\frac{C_{A}^{\prime }/C_{B}^{\prime }}{C_{A\;}/C_{B}}$$

where, *C*_A _ and  *C*_B_ are the initial concentrations of A and B ions in feed solution, respectively. $C_{A}^{\prime }$ and $C_{B}^{\prime }$ are the concentrations of A and B ions in permeate solution, respectively.

As shown in Figure 3d, without GO membranes, the commercial cellulose acetate (CA) substrate membrane showed nearly no separation ability as the *α*_K/Sr_, *α*_K/U_, and *α*_Sr/U_ (U presents UO_2_^2+^ in this article) are only 1.6, 1.7, and 1.1, respectively. Once the GO‐M1 membrane was used, the *α*_K/Sr_, *α*_K/U,_ and *α*_Sr/U_ increased to 5.1, 11.3, and 2.2, indicating the sieving effect of GO membranes on ions with various sizes. As expected, the separation performance improved with a narrowed interlayer spacing *d* of GO membranes. Through reducing *d*, the *α*_K/U_ continuously increased from 11.3 (GO‐M1) to 15.3 (GO‐M2), 36.5 (GO‐H1), and 249.4 (GO‐H2). It should be noted that for GO‐H2, as a cut‐off blocking of UO_2_^2+^ was achieved, the detection limit was used as the UO_2_^2+^ concentrations in permeate solution. In the literatures, the separation coefficients for monovalent and divalent ions are usually only about 3–16,^[^ ^48^, ^49^ ^]^ so the K^+^/UO_2_^2+^ separation performance of GO‐H2 is about 15–80 times higher than that of other graphene‐based membranes. For Sr^2+^ and UO_2_^2+^, a distinct increase of *α*_Sr/U_ from 2.2 (GO‐M1) to 2.7 (GO‐M2), 6.0 (GO‐H1) and 20.5 (GO‐H2) was observed. Additionally, we confirmed that the separation performance has little to do with the adsorption effect (Figure S9, Supporting Information). These results suggested that the GO membranes could be used for the UO_2_^2+^ sieving in high acidic solutions.

### 2.4 Influence of Acidity on the Separation Performance of GO‐H2 Membranes

**Figure 4:**
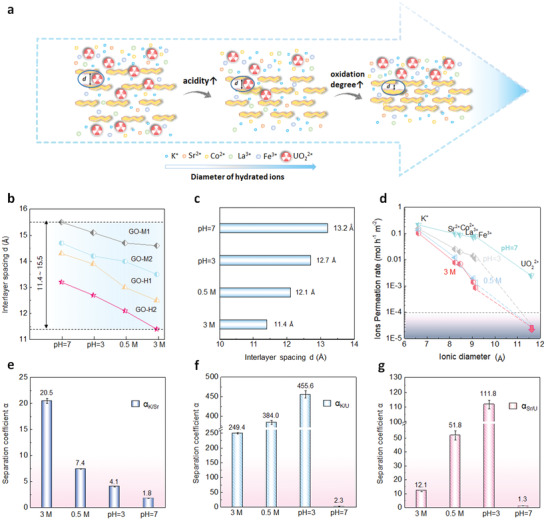
Effect of acidity on GO membranes permeation performance. a) Dual‐adjustment on the interlayer spacing *d* through controlling the solution acidity and the oxidation degree of GO. b) Adjustment range of *d* by dual‐adjustment method. c) Interlayer spacing for GO‐H2 soaked in aqueous solutions with various acidity (pH = 7, pH = 3, 0.5, and 3 mol L^−1^) for 5 h. d) Ion permeation tests in different concentration nitric acid after 5 h for GO‐H2. Gray area stands for the below‐detection limit of our measurements with the arrow indicating the limit of ions. e–g) Separation coefficient *α*_K/Sr_, *α*_K/U_, and *α*_Sr/U_ for GO‐H2 under different acidity.

The dual‐adjustment strategy of combining acidity with oxidation degree exerted a confinement to the GO membrane, preventing it from swelling and reducing the interlayer spacing to below the hydrated diameter of UO_2_^2+^ ions (**Figure** **4**a). Figure 4b shows interlayer spacing can be precisely controlled in a wide range from 15.5 to 11.4 Å through this dual‐adjustment strategy. GO‐H2, as the optimal sample with highest oxidation degree, had the widest control range (13.2–11.4 Å) (Figure 4c). Therefore, the separation performance of GO‐H2 in various acidity conditions were investigated. As shown in Figure 4d, the reduction degree of permeation rates was also relevant to the size of the ions. The permeate rates of K^+^ (6.62 Å), Sr^2+^ (8.24 Å), UO_2_^2+^ (11.6 Å) reduced about 2, 12, 165 times separately with acidity increasing from pH = 7 to 3 mol L^−1^ HNO_3_. As a result, the separation coefficient of K^+^ and Sr^2+^ (Figure 4e) increased from 1.8 to 4.1, 7.4 and 20.5, when the acidity raised from pH = 7, pH = 3, 0.5 and 3 mol L^−1^ HNO_3_.

Particularly, for the sieving of UO_2_^2+^, the cut‐off blocking of UO_2_^2+^ ions was achieved under all acidic conditions from pH = 3 to 3 mol L^−1^ HNO_3_. It can be ascribed to the effective nano channels *μ* of GO‐H2 decreasing from 9.8 to 9.3, 8.7, and 8.0 Å. Therefore, the separation coefficients *α*_K/U_ and *α*_Sr/U_ in pH = 3, 0.5 and 3 mol L^−1^ HNO_3_ were calculated using detection limit of uranium and concentration of potassium and strontium. As shown in Figure 4e, *α*_K/U_ showed a dramatic increase from 2.3 (pH = 7) to 249.4 (3 mol L^−1^ HNO_3_). A further increase of *α*_K/U_ to 384.0 (0.5 mol L^−1^ HNO_3_) and 455.6 (pH = 3) was also observed, which can be explained as that the permeance of K^+^ and Sr^2+^ were improved by expanding the interlayer spacing. The improvement of *α*_Sr/U_ from 1.3 (pH = 7) to 12.1 (3 mol L^−1^ HNO_3_), 51.8 (0.5 mol L^−1^ HNO_3_) and 111.8 (pH = 3) was also observed (Figure 4f). These results manifested that by precisely controlling the interlayer spacing of GO membrane, it could not only realize the blocking of UO_2_^2+^, but also obtain a high permeation rate of other ions, which are the two vital factors to achieve an excellent separation performance. GO‐H2 had excellent sieving performance in low‐acid and high‐acid environments, showing a great potential for application in other systems.

## 3 Conclusion

In summary, we have showed the possibility to precisely control the interlayer spacing *d* of GO membrane by adjusting the acidity of solution and the oxidation degree of GO nano sheets, which might be useful in many sieving applications. Through the dual‐adjustment strategy, the interlayer spacing of GO membranes can be precisely controlled in a wide range from 15.5 to 11.4 Å, and sieving uranyl ions from other nuclide ions was successfully realized in pH = 3–3 mol L^−1^ nitric acid aqueous solution. It indicates that our work provides a kind of novel GO membrane and separation method for the uranium separation in high acidic solution, which could be hardly realized by other separation methods or materials. Our work has also broadened the application field of the GO membrane for metal ions separation in radioactive liquid waste, opening a new research area. The GO membrane has potential to facilitate the future development of separating nuclide ions with various sizes from radioactive liquid waste in large‐scale.

## 4 Experimental Section

##### Synthsis of GO Dispersions

Graphene oxide (GO) was prepared by modified two steps Hummers’ method. 200 mesh natural graphite oxide flakes (Alfa Aesar), sulfuric acid (Beijing Chemical works), and phosphorus pentoxide (Beijing Chemical works) were used for synthesizing preoxidized GO. Then, they were further oxidized in the sodium nitrate (Beijing Chemical works) and potassium permanganate (Aladdin Industrial Corporation) under low temperature (less than 20 °C), and further oxidized under middle temperature (35 °C) and high temperature (90 °C). Then, the solution was diluted with distilled water, followed by adding 30% hydrogen peroxide, purified in dialysis tubes for a week, and dried through freeze drying method. GO was exfoliated in distilled water for 5 h by bath sonication (80 W), followed by centrifugation, 2 times, at 3500 r.p.m to remove the un‐exfoliated GO, finally GO dispersions with concentrations of about 1 mg mL^−1^ were obtained. Three GO samples with different oxidation degree were obtained by modifying the reaction time for middle and high temperature.

##### Synthsis of GO Membrane

GO membranes were prepared by vacuum filtration of GO suspension through cellulose acetate membranes. After the filtration, GO membranes were dried in the air at room temperature. The cellulose acetate (CA) membranes act as mechanical supports for permeation test. These support membranes with an average pore size of 0.22 µm did not show any impact on the permeability by comparison. The thickness of the GO membranes was about 800 nm.

##### Ion‐Penetration Test

A H‐shaped glass filter with two compartments (feed and permeate) used in permeation tests. The solution in feed side (80 mL) was prepared by dissolving the solid powder of KNO_3_, Sr(NO_3_)_2_, Co(NO_3_)_2_, La(NO_3_)_3_, Fe(NO_3_)_3_, and UO_2_(NO_3_)_2_ in various nitric acid solutions (pH = 7, pH = 3, 0.5, and 3 mol L^−1^). The concentration of each cation in the feed side was 0.05 mol L^−1^ while the concentration of UO_2_^2+^ was 0.001 mol L^−1^. The permeate side includes 80 mL corresponding nitric acid solutions. Cellulose acetate (CA) membrane with pore size of 0.22 μm used as the support and the effective diameter of GO membrane was 1.6 cm. In ion permeation tests, the initial and 5 h concentration of the permeate compartment was measured through ICP‐OES. 0.5 mL solution at 0 and 5 h was removed from the permeate solution, and then were diluted 20 times. The following equation was used to calculate the permeate rates *J*.

(2) $$J\;=\frac{\left(C_{t}-C_{0}\right)V}{t\times A_{\mathrm{eff}}}$$

where, *C_t_* and *C*_0_ are the initial and 5 h concentrations of metal ions in permeate solution, respectively (mol L^−1^). *t* is the permeate time (5 h), *A*_eff_ is the effective area of GO membrane *A*_eff_  =   *πr* ^2^  =   2.01 cm^2^.*V* is the volume of the feed and permeate solution (80 mL). In addition, to eliminate the influence of initial metal ions concentration, separation coefficient *α*_A/B_ was used for estimate the separation performance in this paper.

##### Materials Characterizations

The electronic images of the GO membranes were observed via a field‐emission SEM at high vacuum with an accelerating voltage of 15 kV. FT‐IR spectra were investigated on an infrared spectrometer (Bruker, Horiba, Germany). Raman spectra were investigated on a Raman spectrometer (Evolution, Horiba, France). The interlayer spacing of dry and solvent‐soaked GO membranes were carried out on X‐ray diffraction (XRD, Cu K*α* radiation) in the 2*θ* range from 2° to 30° with an interval of 0.02°. XPS spectra were taken out by using a X‐ray photoelectron spectrometer (PHI Quantro SXM, ULVAC‐PHI, Japan). Atomic force microscope (AFM) was used to detect the size and height of the nano GO flakes (SPM‐960, Shimadzu, Japan). Inductively coupled plasma‐optical emission spectrometer (ICP‐OES) was used to detect the concentration of ions (AR10S‐SOP, Spectro, Germany).

## Conflict of Interest

The authors declare no conflict of interest.

## Supporting information

Supporting Information
