# Supplementary material for: Graphene Oxide Membranes for Tunable Ion Sieving in Acidic Radioactive Waste
Source: Adv Sci (Weinh). 2021 Feb 18;8(7):2002717. doi: 10.1002/advs.202002717 (PMC8025005; doi:10.1002/advs.202002717)
Supplement: Supplementary file 1 — Supporting Information [file ADVS-8-2002717-s001.pdf]

WILEY-VCH

© 2020 Wiley-VCH GmbH

ADVANCED SCIENCE Open Access

Supporting Information

for *Adv. Sci.,* DOI: 10.1002/advs.202002717

Graphene Oxide Membranes for Tunable Ion Sieving in Acidic Radioactive Waste

Tong Wu, Zhe Wang, Yuexiang Lu, Shuang Liu, Hongpeng Li, Gang Ye, and Jing Chen**

Supporting Information

Graphene Oxide Membranes for Tunable Ion Sieving in Acidic Radioactive Waste

Tong Wu, Zhe Wang, Yuexiang Lu, Shuang Liu, Hongpeng Li, Gang Ye, and Jing Chen**

T. Wu, S. Liu, H. P. Li, Prof. Y. X, Prof. G. Ye, Prof. J. Chen

Institute of Nuclear and New Energy Technology

Tsinghua University

Beijing 100084 (P. R. China)

E-mail: luyuexiang@mail.tsinghua.edu.cn & jingxia@mail.tsinghua.edu.cn

Z. Wang

The MOE Key Laboratory of Resource and Environmental System Optimization

School of Environment and Chemical Engineering

North China Electric Power University

Beijing 102206 (P. R. China)

* Correspondence to: luyuexiang@mail.tsinghua.edu.cn; jingxia@mail.tsinghua.edu.cn

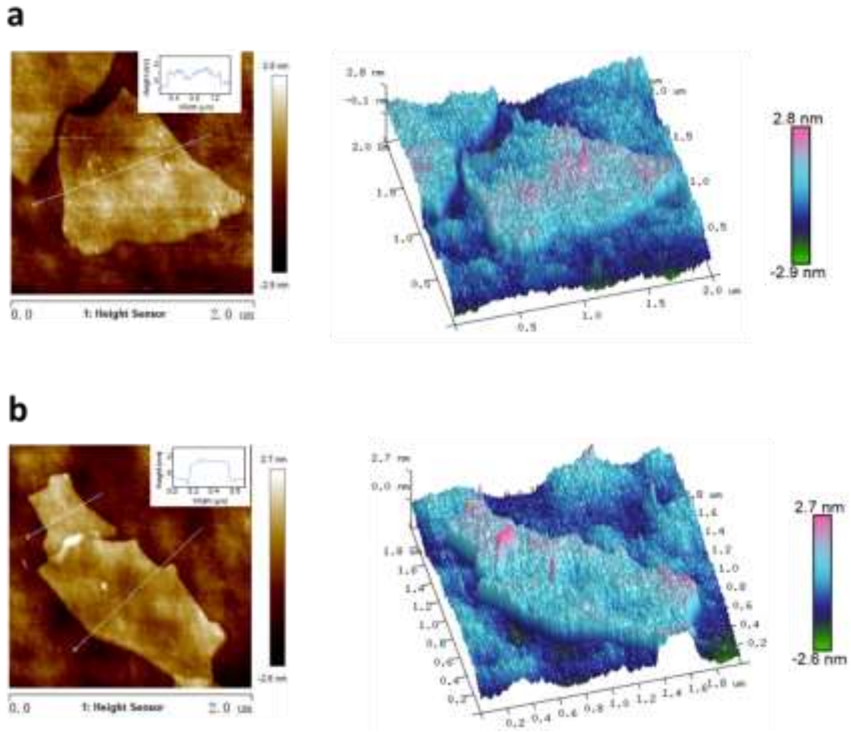

Figure S1. AFM images of GO nanosheets.

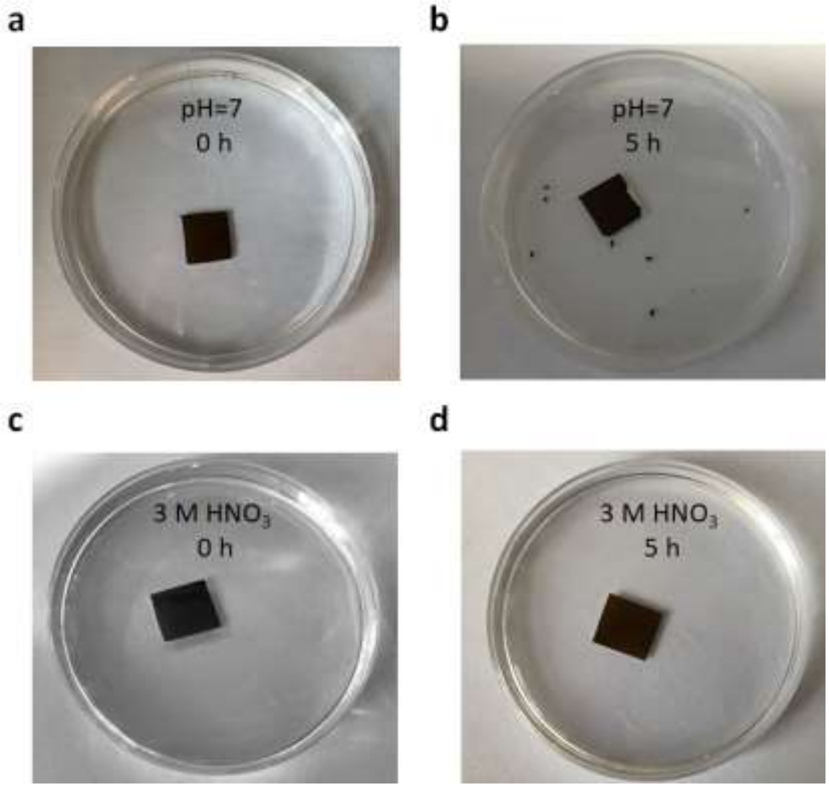

Figure S2. Initial prepared GO membranes soaked in water and 3 M HNO_3 for 5 h.

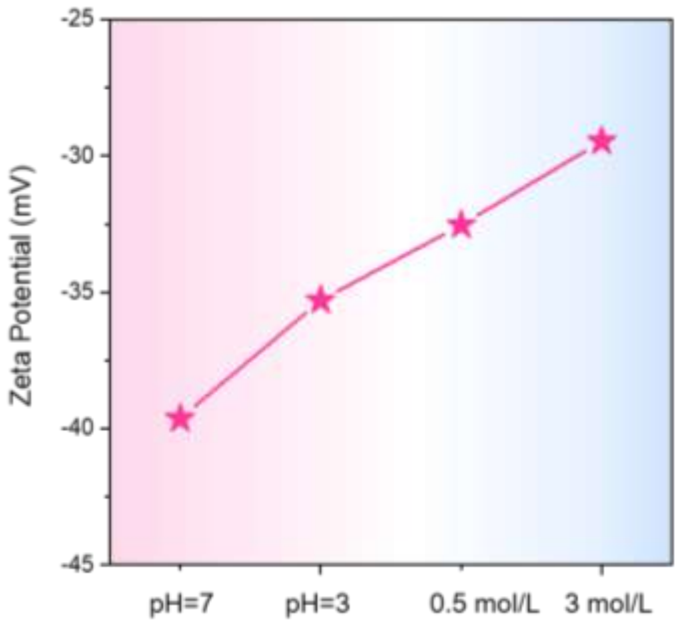

Figure S3. Zeta potential of GO membrane under different acidity.

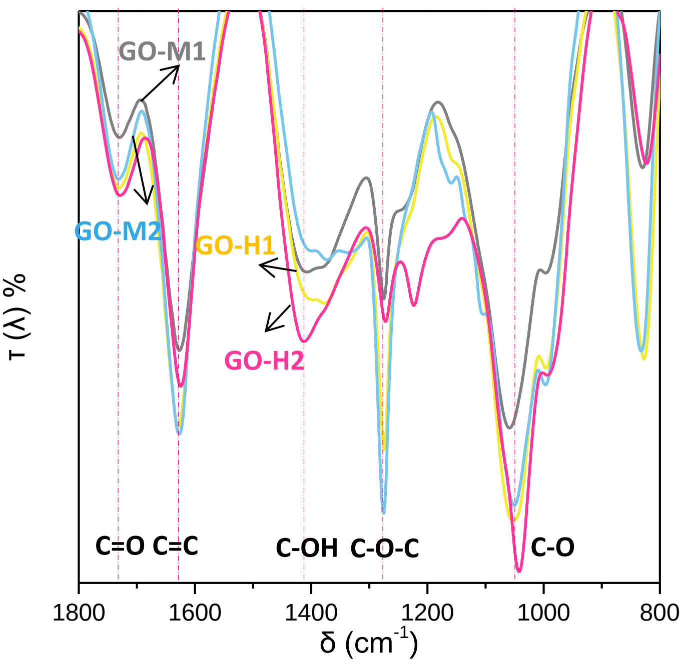

Figure S4. FT-IR spectra of four GO membranes with different oxidation degrees.

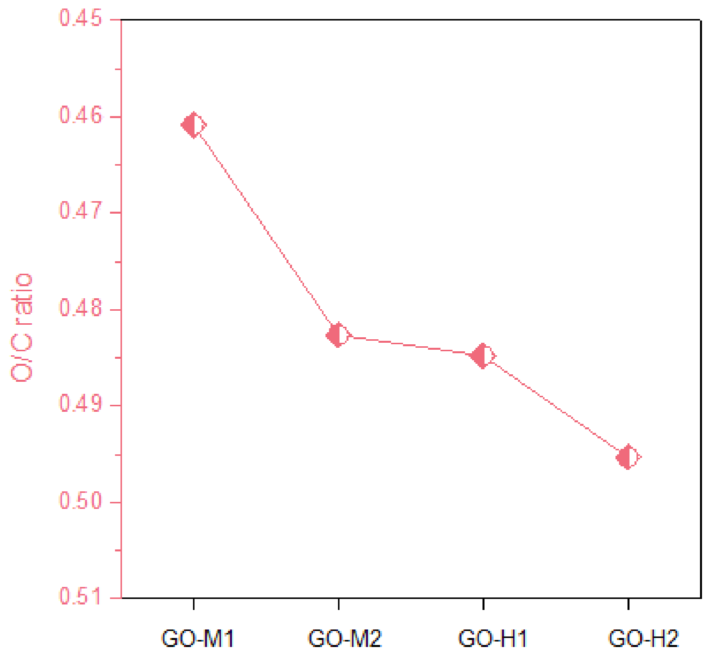

Figure S5. The ratio of O and C for four GO membranes measured by XPS.

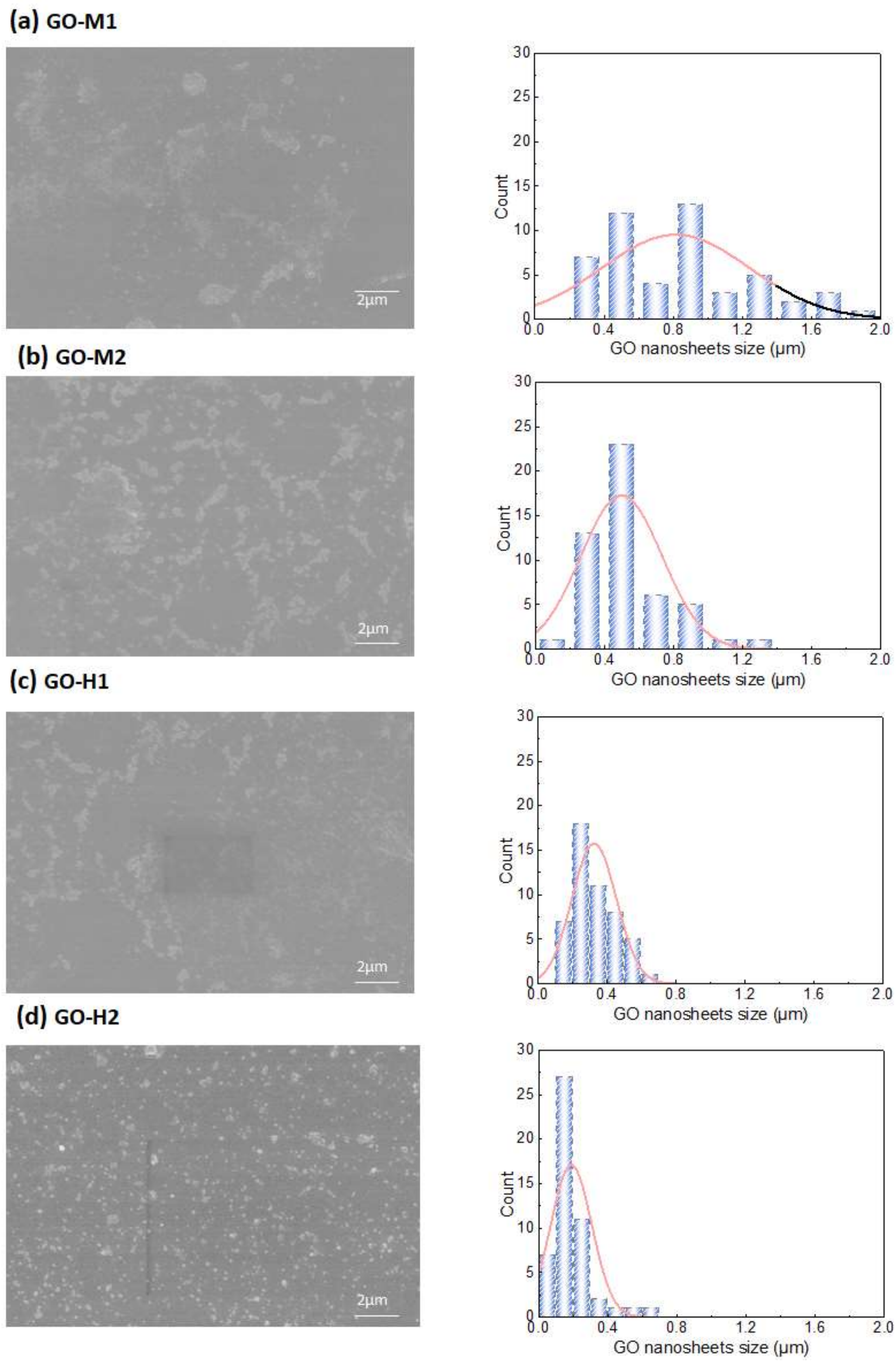

Figure S6. SEM images of four kinds of GO nanosheets and corresponding size statistics.

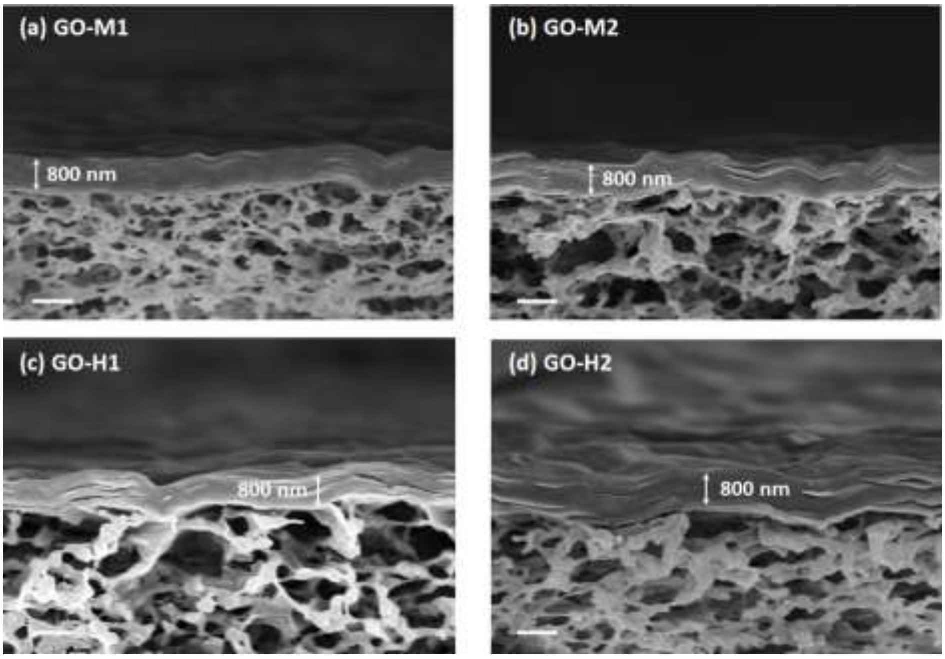

Figure S7. Cross-sectional morphologies of four GO membranes. Scale bar, 1 μm.

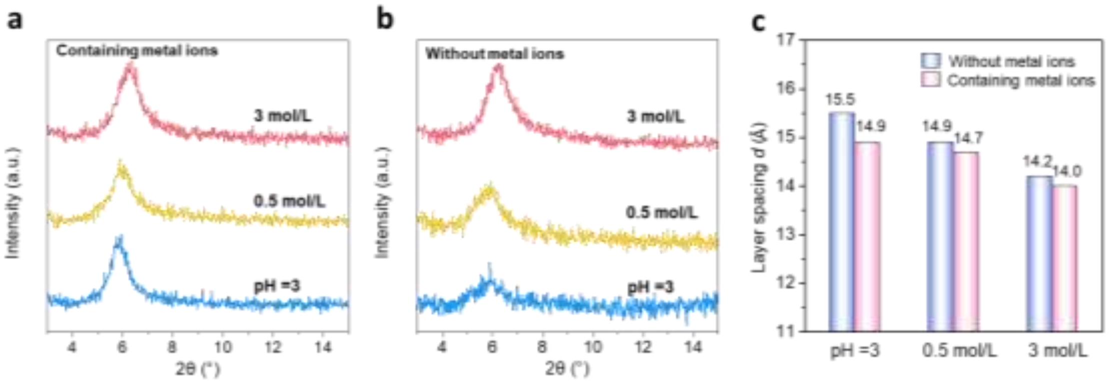

Figure S8. XRD pattern of GO membranes (GO-M2) soaked in different nitric acid for 5 h and the corresponding interlayer spacing.

The interlayer spacing was tested after soaked in the solution containing metal ions (K^+, Sr^{2+}, Co^{2+}, La^{3+}, Fe^{3+}). We found that the interlayer spacing also decreased as the acidity increasing, showing that with the existence of metal ions our control method is still working. We also found that the metal ions do have some influence on the interlayer spacing. Under the same acidity, the interlayer spacing of GO soaked in solution with metal ions is smaller than that without metal ions. Furthermore, the reduced value of *d* in these two conditions gradually decreases as the acidity increasing. At pH=3, the *d* of GO-M2 was reduced by 0.6 Å at the presence of metal ions, while in pure nitric acid solution (3 mol/L HNO_3) the reduction of *d* was only 0.2 Å after metal ions were added. These results showed that the influence of metal ions decreased with the acidity increasing, suggesting that the acidity played a more important role in adjusting the *d*. Firstly, the concentration of metal ions in this work (≤0.05 mol/L) was lower than that in other literatures (0.1 mol/L) that utilizing metal ions to adjust the *d* [1]. And secondly, under high acid conditions, the influence of protons on the EDL may be greater than metal ions as the concentration of protons is one order higher than that of metal ions. In fact, in the separation experiments, as the initial concentration and species of mixed ions in the feed solution were all the same, the different separation performance was only caused by the different acidity.

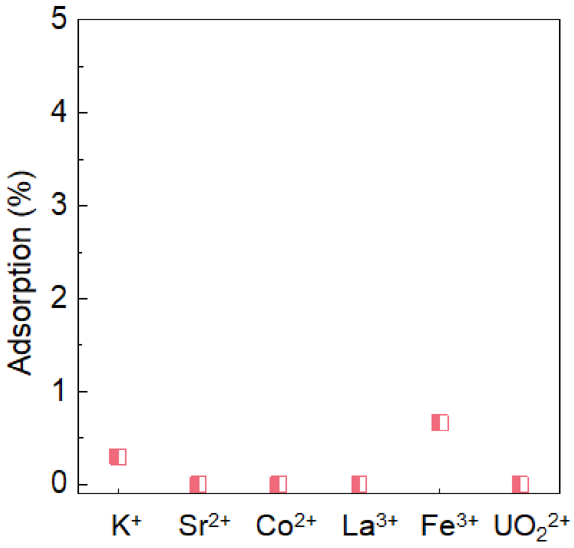

Figure S9. Adsorption of GO-H2 membrane to various cations under 3 mol/L HNO_3.
